# A soluble guanylate cyclase activator protects from diabetic nephropathy beyond standard of care in the ZSF1 rat

**DOI:** 10.1186/2050-6511-16-S1-A4

**Published:** 2015-09-02

**Authors:** Steven S Pullen, Kathleen A Lincoln, Paul C Harrison, Hongxing Chen, Hong Wang, Holly Clifford, HuSheng Qian, Diane Wong, Chris Sarko, Jehrod Brenneman, Ryan Fryer, Jeremy Richman, Glenn A Reinhart, Carine Boustany

**Affiliations:** 1 CardioMetabolic Disease Research and Medicinal Chemistry, Boehringer Ingelheim Pharmaceuticals Ridgefield, CT, USA

## 

The pathogenesis of diabetic nephropathy is associated with abnormalities of renal nitric oxide generation and signaling. We evaluated the effect of BI 684067, a soluble guanylate cyclase (sGC) activator, in combination with the current standard of care (SoC), on the progression of diabetic nephropathy. Male ZSF1 rats were administered enalapril (3 mg/kg in drinking water) for 10 days, after which they were randomized to either continue to receive enalapril alone or the combination of enalapril and one of three doses of BI 684067 (20, 40 and 80 mg/kg) in chow for 10 weeks. Weekly urinary protein to creatinine ratio (UPCR) as well as daily mean arterial pressure (MAP) and heart rate (HR) were measured. At study end, kidneys were assessed for glomerular lesions and α-SMA expression, a marker of myofibroblast activation. The combination of BI 684067 and enalapril resulted in significant dose-dependent decreases of the following when compared to enalapril alone: UPCR (BI 684067 at 20, 40, and 80 mg/kg : 27, 39, 48% reductions respectively), incidence of glomerulosclerosis (BI 684067 at 20, 40, and 80 mg/kg: 29, 32, 44% reductions, respectively) and α-SMA expression (BI 684067at 20, 40, and 80 mg/kg : 26, 40, 42% reductions, respectively). The MAP was significantly reduced by BI 684067 in combination with enalapril (- 3 mm Hg vs enalapril alone at the doses of 40 and 80 mg/kg), however there was no significant effect on HR. These results support the efficacy of an sGC activator in preventing the progression of diabetic nephropathy when combined with the SoC.

